# Needle exchange programs for the prevention of hepatitis C virus infection in people who inject drugs: a systematic review with meta-analysis

**DOI:** 10.1186/s12954-017-0156-z

**Published:** 2017-05-17

**Authors:** Stephen M. Davis, Shay Daily, Alfgeir L. Kristjansson, George A. Kelley, Keith Zullig, Adam Baus, Danielle Davidov, Melanie Fisher

**Affiliations:** 1 School of Medicine, Department of Emergency Medicine, Robert C. Byrd Health Sciences Center, West Virginia University, PO Box 9149, Morgantown, WV 26506-9149 USA; 2 Department of Social and Behavioral Sciences, West Virginia University, PO Box 9190, Morgantown, WV 26506 USA; 3 Department of Biostatistics, West Virginia University, PO Box 9190, Morgantown, WV 26506 USA; 4 Department of Medicine, Section of Infectious Diseases, West Virginia University, PO Box 9163, Morgantown, WV 26506 USA

**Keywords:** Needle exchange program, Meta-analysis, Systematic review, Hepatitis C, Injection drug use, Opioids, Heroin, Pain killers, Pain

## Abstract

**Background:**

Previous research on the effectiveness of needle exchange programs (NEP) in preventing hepatitis C virus (HCV) in people who inject drugs (PWID) has shown mixed findings. The purpose of this study was to use the meta-analytic approach to examine the association between NEP use and HCV prevention in PWIDs.

**Methods:**

Study inclusion criteria were (1) observational studies, (2) PWIDs, (3) NEP use, (4) HCV status ascertained by serological testing, (5) studies published in any language since January 1, 1989, and (6) data available for measures of association. Studies were located by searching four electronic databases and cross-referencing. Study quality was assessed using the Newcastle Ottawa (NOS) scale. A ratio measure of association was calculated for each result from cohort or case–control studies and pooled using a random effects model. Odds ratio (OR) and hazard ratio (HR) models were analyzed separately. Results were considered statistically significant if the 95% confidence interval (CI) did not cross 1. Heterogeneity was estimated using *Q* and *I* ^*2*^ with alpha values for *Q* ≤ 0.10 considered statistically significant.

**Results:**

Of the 555 citations reviewed, 6 studies containing 2437 participants were included. Studies had an average NOS score of 7 out of 9 (77.8%) stars. Concerns over participant representativeness, unclear adjustments for confounders, and bias from participant nonresponse and loss to follow-up were noted. Results were mixed with the odds ratio model indicating no consistent association (OR, 0.51, 95% CI, 0.05–5.15), and the hazard ratio model indicating a harmful effect (HR, 2.05, 95% CI, 1.39–3.03). Substantial heterogeneity (*p* ≤ 0.10) and moderate to large inconsistency (*I* ^*2*^ ≥ 66%) were observed for both models.

**Conclusions:**

The impact of NEPs on HCV prevention in PWIDs remains unclear. There is a need for well-designed research studies employing standardized criteria and measurements to clarify this issue.

**Trial registration:**

PROSPERO CRD42016035315

**Electronic supplementary material:**

The online version of this article (doi:10.1186/s12954-017-0156-z) contains supplementary material, which is available to authorized users.

## Background

### Rationale

Globally, over 184 million people (>2.8% of the world’s population) have been infected with the hepatitis C virus (HCV) [1]. HCV is a blood-borne virus that infects the liver. Approximately 75% of acute HCV infections become chronic [2]. Chronic HCV infection significantly increases the risk of liver disease, especially cirrhosis and liver cancer [1–7]. A majority of the 350,000 deaths attributed to HCV infection each year are caused by cirrhosis and hepatocellular carcinoma [7]. In most countries, the annual incidence of HCV infection has peaked with the exception of Russia where new cases are still increasing [1]. However, a troublesome pattern of new HCV cases observed in the USA suggests another or recurrent public health epidemic [1, 2, 8, 9].

The incidence of HCV in the USA declined from 2000–2005 and plateaued during 2005–2010 [8]. Thereafter, the number of reported acute cases increased significantly with a 2.6-fold increase observed between 2010 and 2014 [8]. Collectively, a 364% increase in HCV cases among persons aged ≤30 years was observed in Central Appalachia (Kentucky, Tennessee, Virginia, and West Virginia) between 2006 and 2012 [10]. As a result, the USA has set a goal of reducing new hepatitis C infections from 0.28 cases per 100,000 to 0.25 cases per 100,000 (Healthy People 2020 Objective IID-26) [11].

HCV-infected patients consume a large proportion of healthcare resources in the USA. Between 2001 and 2010, HCV-infected individuals accounted for almost 3 million outpatient, inpatient, and emergency department visits in the USA [6]. Estimated healthcare costs associated with the treatment and care of chronic HCV was $6.5 billion in 2011 and is expected to peak at $9.1 billion in 2024 [3].

Although HCV can be transmitted in several ways, the primary mode of HCV exposure is percutaneous with injection drug use remaining the largest risk factor for HCV infection [2, 3, 8, 9, 12–18]. People who inject drugs (PWID) account for approximately 60–70% of the incidence of new HCV infections in the USA and many other countries [2]. Globally, it is estimated there are 10 million PWIDs that have HCV infection [18]. The prevalence of HCV infection in PWIDs ranges between 40 and 90% and has been observed to be as high as 98% [10]. Recent evidence from the USA has shown that many of these PWIDs are White [2, 9, 14, 15] and young (<35) [2, 8, 9, 14, 15] and have a history of prescription drug use and abuse, especially prescription opiates [2, 13–15]. Furthermore, two recent studies suggest that HCV-infected PWIDs are more likely to reside in non-urban areas [14, 15].

Harm reduction interventions aim to reduce individual and societal harms stemming from drug use by targeting risky behaviors and risky settings [19]. A needle exchange program (NEP) is one popular harm reduction intervention that seeks to reduce risky settings. NEPs provide clean needles in exchange for used needles to minimize the reuse of needles contaminated with infectious disease during drug injection [19]. Many NEPs also provide other prevention materials and services such as additional sterile injecting supplies (e.g., cotton and alcohol swabs), wound care and safe injecting practices education, and linkage and referral to substance treatment programs for those PWIDs ready to quit injecting [20]. However, the evidence for the effectiveness of NEPs in preventing HCV among PWIDs is mixed [21]. For example, a systematic review with meta-analysis of several interventions, including NEPs, to prevent HCV infection in PWIDs observed an increased risk of HCV seroconversion associated with NEP use (RR 1.62, 95% CI, 1.04–2.52), although substantial heterogeneity was observed (*Q* = 32.3; *P* < 0.01; *I* ^*2*^ = 81%) [12]. Furthermore, this systematic review only included studies published through April 2010, and selected studies were limited to the injection of illegal drugs (heroin, amphetamine, and cocaine) by PWIDs. Thus, studies describing the injection of prescription opioids were not considered for inclusion in the analysis. In another study, a review of reviews by MacArthur et al. [21] concluded that there was insufficient evidence to support or discount the effectiveness of NEPs for the prevention of HCV in PWIDs. A more recent systematic review of needle/syringe programs for the reduction of HCV infection among PWIDs by Abdul-Quader et al. [22] found that 6 of the 15 included studies (40%) reported decreases in HCV infection. However, included studies only examined structural and population level interventions, as opposed to the association between individual use of NEPs and HCV infection. Finally, a recently published systematic review with meta-analysis by Sawangiit et al. [23] examined the effectiveness of pharmacy-based NEPs for PWIDs. However, in addition to specifically focusing on pharmacy-based NEPs, which may not always exchange needles [24], this study only examined the impact of these programs on the prevalence of HCV versus the prevention of new infections (incidence). To the best of the authors’ knowledge, no other systematic reviews with or without meta-analyses of the effectiveness of NEPs in preventing HCV in PWIDs currently exist. Therefore, the primary objective of this study was to conduct a systematic review with meta-analysis to examine the association between NEPs and the prevention of HCV in PWIDs.

## Methods

### General procedure

The conduct and reporting of this study followed the recommended guidelines from the Preferred Reporting Items for Systematic Reviews and Meta-Analysis (PRISMA) statement [25]. This systematic review with meta-analysis is registered in the PROSPERO trial registry (CRD42016035315).

### Study eligibility criteria

The a priori inclusion criteria for this systematic review with meta-analysis were as follows: (1) observational studies, (2) PWIDs, (3) NEP use, (4) HCV status ascertained by serological testing (saliva or serum), (5) studies published in any language after January 1, 1989, and (6) data available or calculable for measures of association between participation in a NEP and HCV infection. Studies were excluded based on inappropriate study design, population, intervention, or lack of available information to calculate a measure of association between program participation and HCV infection.

Although randomized controlled trials are considered the highest level of evidence for examining the effect of an intervention on a health outcome, [26] it was anticipated that no such trials would be found given that it would be highly unethical (violation of the ethical principle of *beneficence*) to randomize subjects away from a potentially beneficial treatment (i.e., needle exchange). Therefore, the focus of the review was on observational studies.

An a priori decision was made to exclude studies that reported the use of supervised injection facilities (SIFs). The rationale for this exclusion was based on the observation that while SIFs provide a safe environment for drug users to inject drugs, such facilities may not always provide (exchange) clean needles [27]. Additionally, studies describing the distribution of clean syringes from pharmacies were also excluded because such programs typically involve the sale of clean syringes with or without a prescription but may not involve the exchange of clean needles for dirty needles [24]. HCV status ascertained by serological testing was chosen as the primary outcome because previous research has demonstrated low sensitivity when HCV status is self-reported by PWIDs [28]. The year 1989 was chosen as a starting point for the search because this was the year that the HCV antibody was identified, and thus, enabled serological testing to detect the virus [29]. Based on the recommendations by van Driel et al. [30], no searches for unpublished works such as dissertations and conference abstracts or other unpublished reports were conducted.

### Data sources

The following databases were searched between July 18, 2016, and August 24, 2016: (1) PubMed, (2) Scopus, (3) Web of Science, and (4) CINAHL. The search strategy and terms were based on the work of the HCV synthesis project [29] and was modified to include specific search terms related to NEP that were used by Abdul-Quader et al. [22] in their systematic review of population level outcomes following the implementation of NEP. Additionally, the term “people who inject drugs” was included in the search given the observation by the authors that this term has been commonly used to refer to injection drug users in more recent research literature. Although there was slight variation in the specific search format between databases, the following search terms and combinations were used: (hepatitis C OR HCV) AND (intravenous drug abuse OR intravenous drug use OR drug misuse OR drug addict OR injecting drug use OR drug abuse OR people who inject drugs OR PWID OR PWID) AND (prevention OR risk factor OR epidemiology OR prevalence OR incidence OR seroprevalence OR seroincidence OR seroconversion OR genotype OR coinfect*) AND (needle exchange OR needle exchange program OR syringe exchange program OR syringe access program) AND (“1989/01/01” [Date - Publication] : “3000” [Date - Publication]). Search strategy examples for the four databases searched are included in Additional file 1. In addition to database searches, cross-referencing from retrieved studies and reviews was also conducted. After identifying the final number of studies to be included, the number needed to read (NNR) was calculated by taking the inverse of the precision, which was defined as the number of included studies divided by the total number of studies screened after removal of duplicates [31]. All studies identified during the search were stored in EndNote® version 7.4 [32].

### Study selection

Two researchers (SMD and SD) independently reviewed studies for selection and abstracted data from eligible studies with discrepancies resolved by consensus and discussion with a third researcher (GK), if needed. Duplicate studies were discovered by using the “Find Duplicates” tool in EndNote® 7.4 [32] and by manual examination. After removal of duplicate studies, abstracts of all studies were reviewed and the full text of studies appearing to meet the inclusion criteria were obtained and reviewed. Studies that met all of the inclusion criteria were selected. The authors were not blinded to journal titles and study authors and their associated institutions during the review. Reasons for exclusion from further review were coded as one or more of the following: (1) inappropriate population (i.e., not PWIDs), (2) inappropriate intervention (i.e., not a NEP), (3) inappropriate comparison (i.e., no comparison to non-exchange users), (4) inappropriate outcome (i.e., self-reported HCV status), and (5) lack of data to enable calculations of the association between program use and HCV infection.

### Data abstraction

A codebook containing 85 items was developed a priori using Microsoft Excel 2013®, [33] and is included in Additional file 2. The major categories of variables coded by the authors were based upon the HCV Synthesis Project [29] and included (1) study characteristics (author, journal, year, funding status, design, inclusion criteria, recruitment method, recruitment locations, method of determining PWID status, specimen type, and HCV test method), (2) participant characteristics (age, gender, ethnicity, duration of drug use, type of drug used, frequency of use), and (3) outcome characteristics (prevalence, incidence, number of person years, sample size, and variables adjusted for, if applicable). The primary outcome of this study, established a priori, was the association between HCV seroconversion and use of a NEP.

### Risk of bias assessment

The risk of bias in selected studies was assessed using the Newcastle-Ottawa Quality Assessment Scale (NOS) [34]. Consistent with previous research, no study was excluded based on the risk of bias assessment [35].

### Statistical analysis

The a priori plan was to conduct an aggregate data meta-analysis with the study as the unit of analysis.

#### Calculation of effect sizes

The primary outcome for this study was the association between HCV seroconversion and participation in a NEP observed in either cohort studies that follow seronegative individuals over time to monitor seroconversion or case–control studies. This outcome was calculated as the log odds ratio (OR) or the log hazard ratio (HR). Because hazard ratios include a time component, ORs and HRs were analyzed separately.

Where possible, published ratios (OR or HR) and confidence limits from individual studies were used to calculate the log ratios and corresponding logs of the standard errors. If associations in individual studies were not presented in ratios, only log odds ratios were calculated using the reported number of HCV infections and the total number of participants in each group (NEP users and non-users). Missing log hazard ratios were not calculated due to the unavailability of time data. If an exact *p* value was reported instead of a confidence interval (CI), the standard error was calculated using the following formula [26]: log(OR)/z. If reported, adjusted effects were used as the primary outcome under the assumption that such effects have been adjusted for potential bias in the observed association between NEP participation and HCV infection. For ease of interpretation, log ratios were converted back to odds ratios and hazard ratios after analysis.

#### Pooling estimates

Effect size estimates from individual studies were pooled using a random effects model [36]. Between-study heterogeneity was evaluated using the *Q* statistic, and the percentage of variation in effect estimates due to heterogeneity was assessed using the *I* ^*2*^ statistic [37]. Based on current recommendations, heterogeneity was considered to be substantial if the *p* value for the observed *Q* statistic was ≤0.10 [26]. The amount of heterogeneity present (as assessed by *I* ^*2*^ values) was interpreted according to the following categories: <25% (“very low”); 25 to <50% (“low”); 50 to <75% (“moderate”); and 75% or greater (“large”) [37]. Effect sizes were calculated after each study was removed from the model in order to assess the influence of each study on the overall results. In addition, cumulative meta-analysis, ranked by year of publication, was conducted to examine the accrued results over time. Ninety-five percent confidence intervals that did not cross 1 were considered to be statistically significant with values below one indicative of a decrease in the odds or risk of HCV seroconversion (evidence of a *preventative* or *positive effect*). Values significantly above one were considered to indicate a *harmful* or *negative effect*. Values that crossed 1 were considered to indicate *no effect* from NEP participation on the prevention of HCV infection.

An a priori plan was made to assess small-study effects (publication bias, etc.) using funnel plots and Egger’s regression intercept test (one-tailed). However, we were unable to conduct these analyses because we did not have at least 10 effect sizes, the minimum sample size recommended by Sterne et al. [38]. Similarly, a priori plans to conduct a mixed-effects meta-regression to examine potential covariates and a moderator analysis to examine potential differential study effects from different study designs (e.g., cohort and case study) were not conducted due to insufficient sample sizes (<10 effects) [26]. All analyses were carried out using Comprehensive Meta-Analysis (version 3.0) [39].

## Results

### Study characteristics

**Fig. 1:**
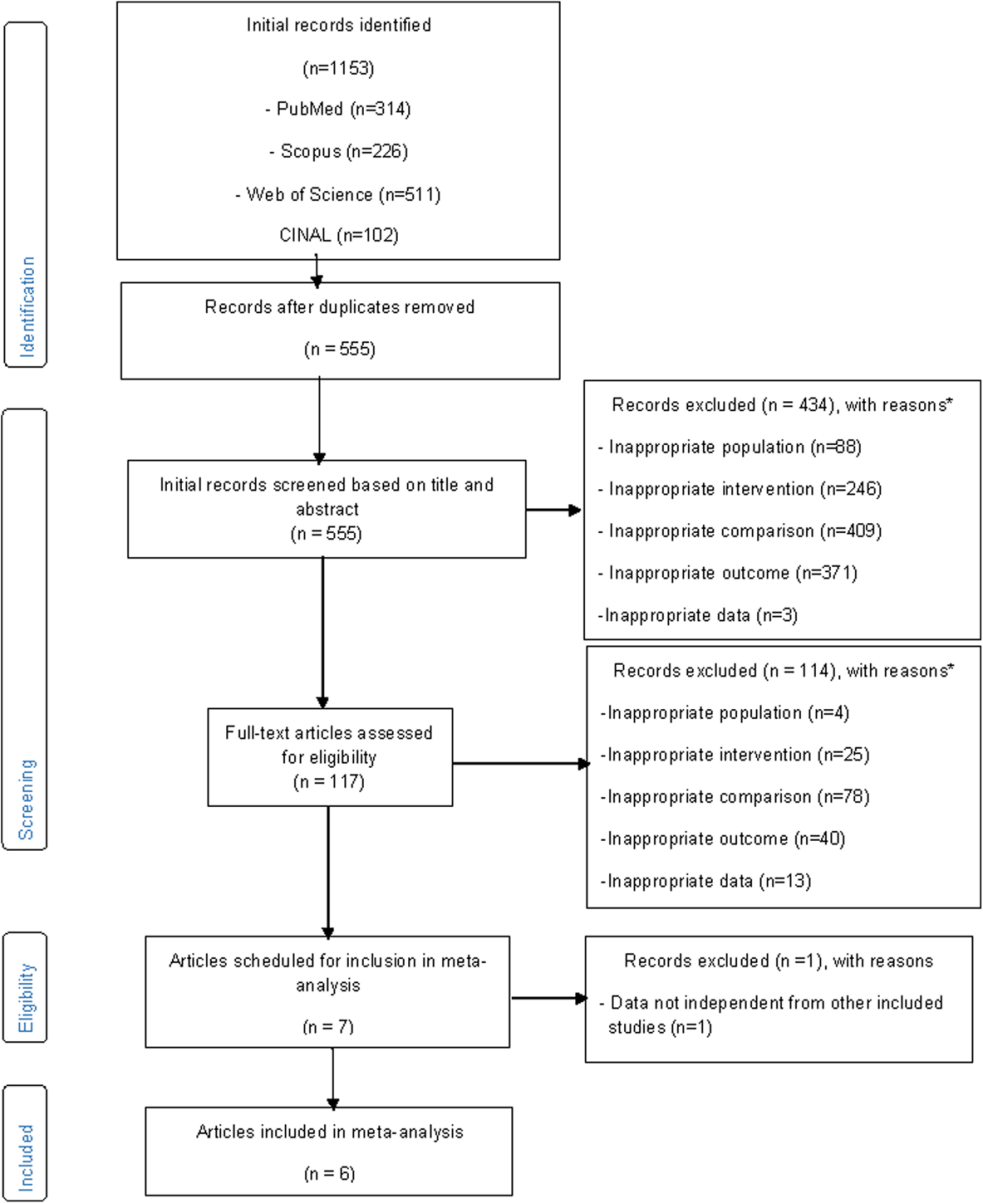
Flow diagram for the selection of studies. *Number of reasons exceeded the number of studies because some studies were excluded for more than one reason

Overall, of the 555 references examined, 6 studies, [40–45] containing data from 2437 PWIDs, were included in the final review and analysis. One study [46] was identified that contained estimates based on data from the same sample of PWIDs collected in the same location during the same time periods as those included in another larger and more recent study that was selected for inclusion [42]. Therefore, this study was eliminated from analysis given that these data would have violated the statistical requirement of independence of effect size estimates. The precision of the search was 1% (6/555), and the NNR was 100. Figure 1 diagrams the search process and includes reasons for the exclusion of various studies from the final analysis. Table 1 lists the general study characteristics. Half of the studies were conducted in the USA [40–42], followed by two conducted in Canada [43, 44], and one conducted in Afghanistan [45]. Without exception, all studies were conducted in densely populated urban locations. All studies were published in the English language. There was one case–control study [40] and five cohort studies [41–45].

**Table 1:** General characteristics of studies

Study	Country	Location	Design	Participants	Intervention	HCV measurement
Hagan 1995 [40]	USA	Tacoma, WA	Case–control	*Cases*: 20 men and women (5 NEP users, 15 non-users), heterosexual injection drug user, have a discrete date of onset of clinical symptoms, serum aminotransferase levels greater than 2.5 times the upper limit of normal, exclusion of other causes of liver injury, serum test positive for hepatitis C *Controls*: 26 men and women (19 NEP users, 7 non-users), current injection drug user from other Tacoma Pierce County Health Department services (including those individuals entering a methadone drug treatment program or attending the HIV testing center) during the time cases were reported (1991–1993)	Ever used NEP	serum
Hagan 2004 [41]	USA	Seattle, WA	Cohort	484 men and women (324 NEP users, 160 non-users), drug injection during previous year, English or Spanish speaking, age 14 years or older, and not already in the study	Use of NEP (yes/no)	serum
Holtzman 2009 [42]	USA	Baltimore, MD Chicago, IL Los Angeles, CA New York, NY	Cohort/RCT^a^	1202 men and women (518 NEP users, 684 non-users) that participated in one of three CIDUS studies: CIDUS I: 18–50 years old, reported injecting drugs in the past 12 months; CIDUS II: 18 to 30 years old, reported injecting drugs in the past six months; CIDUS III: injection drug users 15 to 30 years old, reported injecting drugs in the past 6 months	CIDUS I and II: NEP participation within the past six months; CIDUS III: NEP participation within the past 3 months	serum
Patrick 2001[43]	Canada	Vancover, BC	Cohort	155 men and women (75 NEP users, 80 non-users), PWIDs, residing in the greater Vancouver area, injected at least once in the previous month	NEP attendance at least once per week in the previous 6 months	serum
Roy 2007 [44]	Canada	Québec Ottawa, ON	Cohort^b^	359 men and women, at least one injection in the past 6 months, participated at least twice in SurvUDI between 1997 and 2003	NEP use in previous 6 months	saliva
Todd 2015 [45]	Afghanistan	Kabul	Cohort	191 men, reported injecting drugs within the past 30 days, residing in Kabul, Dari or Pashto speakers	Any NEP use during the previous 3 months	serum

^a^Retrospective analysis of data that included a subset of data from a randomized controlled trial comprised of a cohort who were seronegative at baseline and followed forward in time

^b^Retrospective analysis of banked specimens

### Participant characteristics

All studies enrolled PWIDs who self-reported injections. However, time since last injection prior to enrollment varied between studies with two studies enrolling participants that had injected in the previous month [43, 45], one study enrolling those that injected in the previous 6 months [44], one study enrolling those that had injected in the past year [41], one study containing a mix of participants who injected in the previous 6 months and 1 year [42], and one study not specifying any length of prior injection [40].

Participants were enrolled in a variety of settings ranging from syringe exchange programs, [44] harm reduction programs which provided motivational counseling, washing facilities, medical care, and infectious diseases testing in addition to syringe distribution [45], emergency rooms [40], county health departments [40], jails [41], streets [41–43], social service agencies [41], and areas of known drug user congregation [45].

A variety of sampling schemes were described including respondent driven [42], criterion [40], convenience [44], random [41], and a variant of time-location sampling [45]. One study did not describe the sampling strategy [43]. Study participants were recruited over two decades with the oldest study recruiting during 1991 to 1993 [40] and the most recent study enrolling participants between June 2007 and March 2009 [45].

Of the 2437 PWIDs, 941 reported participation in a needle exchange program (“NEP users”) and 946 participants did not report using a needle exchange (“non-users”). Two studies did not report needle exchange participation for the number of participants who were HCV seronegative at baseline [44, 45]. With the exception of one study that only enrolled males [45], all other studies enrolled both genders. The percentage of male participants in these studies ranged from a low of 42.3% [40] to a high of 73% [44] with an average of 62%. Participants in all studies were generally young (<40 years). However, reporting of age was variable with the average given in one study (31.8 years [44]), medians in two studies (28 years [43, 45]), and age categories in the remaining three studies [40–42]. In these latter three studies, the proportion of all participants less than 34 years of age was at least 65%.

There was a wide variability in the reporting of participant race and/or ethnicity with two studies [44, 45] not reporting any race and/or ethnicity. Of the five studies reporting race and/or ethnicity, “White” race was the most frequently reported by participants, ranging from a low of 49% [42] to a high of 85% [40].

The types of drugs injected varied between studies. The most frequent drugs injected, self-reported by participants in each study, included heroin [41, 43] and cocaine [44]. Three studies [40, 42, 45] did not report a specific type of drug.

Length of time injecting was also widely variable between studies. Three studies reported median injecting durations of 2 years [42, 45] and 7 years [43]. Another two studies partitioned injecting duration into categories. Hagan et al. [40] reported the following categories: HCV positive cases: <5 years (*n* = 7, 35%), 5+ years (*n* = 13, 65%); HCV negative controls: <5 years (*n* = 6, 23.1%), 5+ years (*n* = 20, 76.9%). Hagan et al. [41] reported the following categories (years): ≤1 (*n* = 57, 13%), 1.1 to 2.0 (*n* = 76, 16%), 2.1–5.0 (*n* = 144, 32%), 5.1–10.0 (*n* = 84, 19%), >10.0 (*n* = 93, 20%). Roy et al. [44] reported an average of 10 years of injecting.

Half of the studies did not report any information related to the frequency of injections [40, 44, 45]. In the three studies reporting injection frequency [41–43], the proportion of participants injecting at least once a day averaged 51.03%.

### Intervention characteristics

Participation in a NEP was assessed by self-report in all six studies. The frequency of participation varied between studies due to heterogeneity in the presentation of results. Two studies reported ever (versus never) using a NEP [40, 41]. Two studies reported NEP use in the last 3 months [42, 45]. Three studies reported NEP use in the last 6 months [42–44], with Patrick et al. [43] requiring NEP attendance at least once per week in the past 6 months.

### Risk of bias assessment

Risk of bias assessment results are shown in Table 2. Individual study scores ranged from 5–8 stars, which represented 55–89% of the total number of stars that can be awarded (9 stars). The five cohort design studies [41–45] averaged 7 stars with the lone case–control study [40] receiving 6 stars.

**Table 2:** New-Castle Ottawa Scale Ratings

Study	Selection	Comparability	Exposure/Outcome
Hagan 1995 [40]	★★★	★★	★
Hagan 2004 [41]	★★★★	★★	★★
Holtzman 2009 [42]	★★★★	★★	★
Patrick 2001 [43]	★★★★	★	★★
Roy 2007 [44]	★★★	★	★
Todd 2015 [45]	★★★★	★★	★★

Four of the six studies [41–43, 45] received the maximum number of stars (4) in the selection category. Concerns over the representativeness of participants resulted in the deduction of one star from this category in the remaining two studies [40, 44]. All but two of the studies received the maximum number of stars (2) in the comparability category. Patrick et al. [43] and Roy et al. [45] each received only one star in this category because adjustments for potential confounders were unclear. No studies received the maximum number of three stars in the assessment of the exposure (case–control) or outcome (cohort) category. Potential bias from participant nonresponse and loss to follow-up was a primary weakness for all studies.

### Primary outcome

**Fig. 2:**
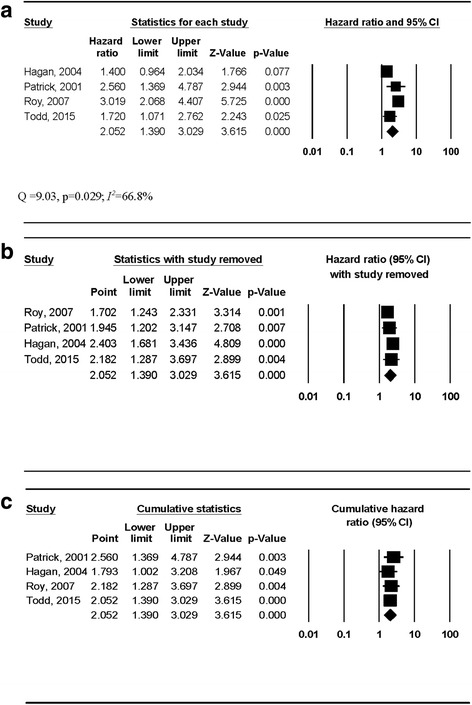
**a** Forest plot for the risk of hepatitis C infection among needle exchange program participants. The *black squares* represent the risk of hepatitis C infection observed in each study with the 95% confidence intervals represented by the lines on each side of the squares. The *diamond* represents the pooled risk of hepatitis C infection with the 95% confidence interval indicated by the left and right extremes of the diamond. **b** Influence analysis for the risk of hepatitis C infection among needle exchange program participants. Influence analysis for point estimate changes in the risk of hepatitis C infection with each individual study deleted from the model once. **c** Cumulative meta-analysis for the risk of hepatitis C infection among needle exchange program participants. The results of each corresponding study, ordered by year of publication from oldest to newest, are pooled with all studies preceding it

**Fig. 3:**
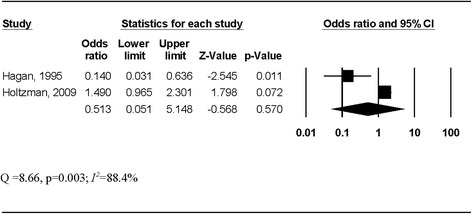
Forest plot for the odds of hepatitis C infection among needle exchange program participants. The *black squares* represent the odds of hepatitis C infection observed in each study with the 95% confidence intervals represented by the *lines on each side of the squares*. The *diamond* represents the pooled odds of hepatitis C infection with the 95% confidence interval indicated by the left and right extremes of the diamond

Study outcomes are shown in Table 3. Four studies reported (or had data enabling calculation of) hazard ratios [41, 43–45], with two studies reporting odds ratios [40, 42]. Three studies [40, 42, 43] adjusted effect estimates for potential confounders. Overall, findings were mixed. A statistically significant harmful effect from participation in NEPs was observed when the four studies that reported hazard ratios were combined (pooled HR, 2.05, 95% CI, 1.39–3.03, Fig. 2a). However, significant heterogeneity and moderate inconsistency were observed (*Q* = 9.03; *p* = 0.029; *I* ^*2*^ = 66.8%). This finding was not influenced by the deletion of any study from the model once (Fig. 2b) and remained consistent over time (Fig. 2c), with all cumulative results yielding confidence intervals that did not cross 1. In contrast, there was no significant association between the odds of HCV seroconversion and participation in a NEP when the two studies that reported odds ratios were combined (pooled OR, 0.51, 95% CI, 0.05–5.15, Fig. 3), although both significant heterogeneity and large inconsistency between studies were observed (*Q* = 8.66; *p* = 0.003; *I* ^*2*^ = 88.4%). Influence analysis and cumulative meta-analysis were not conducted on the odds ratio model given the inclusion of only two studies.

**Table 3:** Study outcomes and adjustments

					95% CI		
Study	Outcome	Adjusted	Adjustments	Estimate	Lower	Upper	*p*
Hagan 1995 [40]	Odds ratio	*y*	Sex, race/ethnicity, duration of drug injecting	0.14	0.03	0.62	
Hagan 2004 [41]	Hazard ratio	*n*		1.40	0.90	1.90	
Holtzman 2009 [42]	Odds ratio	*y*	Sex, age in years, race/ethnicity, education, source of income, site, study time period, injection risk behaviors, and HIV serostatus	1.49	0.96	2.29	
Patrick 2001 [43]	Hazard ratio	*y*	Not described	2.56	1.37	4.79	
Roy 2007 [44]	hazard ratio	*n*		3.02			0.18
Todd 2015 [45]	Hazard ratio	*n*		1.72	1.07	2.76

## Discussion

The primary purpose of this systematic review with meta-analysis was to assess the potential effect of NEP on the prevention of HCV infection in PWIDs. The overall findings were mixed and suggest that NEP could either increase the risk of HCV infection in PWIDs or have no effect. This interpretation is supported by (1) pooled results from studies reporting a hazard ratio that indicate a harmful effect (pooled HR, 2.05, 95% CI, 1.39–3.03), (2) pooled results from studies examining the odds of infection that do not indicate either a preventive benefit or harmful effect (pooled OR, 0.51, 95% CI, 0.05–5.15), and (3) substantial heterogeneity (*p* ≤ 0.10) and moderate (*I* ^*2*^ *= 67%)* to high (*I* ^*2*^ *= 88%)* inconsistency observed for both models. These mixed findings are consistent with previous research.

A review of reviews without meta-analysis by MacArthur et al. [21] of interventions to prevent HCV in PWIDs identified 17 studies with mixed results (9 positive, 2 negative, and 6 no effect) leading to the conclusion that there was insufficient evidence to either support or discount the effectiveness of NEP for preventing HCV.

Hagan et al. [12] conducted a systematic review with meta-analysis of the effectiveness of interventions, including NEP, on the prevention of HCV. Similar to the results from our hazard ratio model, this meta-analysis observed a 62% increase in the risk of HCV seroconversion from participation in syringe access programs (RR, 1.62, 95% CI, 1.04–2.52) with substantial heterogeneity and large inconsistency (*Q* = 32.3, *I* ^*2*^ = 81%). Included studies contained a mixture of no effect (three studies), positive (one study), and harmful (three studies) results. Five of these seven studies were included in our meta-analysis. A single-site study by Thorpe et al. [46] which observed no effect (HR, 1.29, 95% CI, 0.6–2.79) from NEP participation on HCV infection contained data that were also included in the Holtzman et al. [42] multi-site study. Therefore, we excluded this study from the final model to maintain the criterion of independence of effect sizes. Despite extensive searching and multiple electronic and personal queries, we were unable to locate a governmental report by Lamonthe et al. [47] for review and possible inclusion in our systematic review with meta-analysis. Hagan et al. [12] reported that this study demonstrated a harmful effect (HR, 2.24, 95% CI, 1.01–4.98). The current review included one additional cohort study [45], published in 2015, that observed a harmful effect.

A recently published systematic review with meta-analysis of pharmacy-based NEP demonstrated a 74% reduction in the odds of HCV infection (OR = 0.26, 95% CI, 0.18, 0.38) associated with pharmacy-based NEP participation [23]. However, the authors cautioned that this finding was unclear due to the very small number of included studies (*n* = 2) and significant bias concerns. Observed heterogeneity in the study population, and variability in defining the intervention and outcomes reported, further precluded the ability to draw definitive conclusions between HCV infection and pharmacy-based NEP participation. This observation is consistent with the current review. Indeed, the substantial heterogeneity and large inconsistency observed in both the current study and the previous meta-analyses may be related to important between-study differences in the population enrolled, intervention examined, outcome assessed, and type of study design (and associated statistical analyses).

### Variable populations

All studies included in the current review, with the notable exception of Todd et al. [45], enrolled a mix of genders that were largely under the age of 40 and White. However, all studies had slightly different age requirements with some studies enrolling PWIDs as young as 14 years [41] and 15 years [43] compared to other studies that did not report a limit on age [40, 44], only enrolled adults (≥18 years [45]), or had varying age range requirements (e.g., 18–30 and 18–40) [42]. Furthermore, there were a variety of settings in which PWIDs were recruited over an almost 20-year timeframe. However, with the exception of Todd et al. [45], all included studies were conducted in North America. Importantly, no studies took place in the rural, Central Appalachian region of the USA, an area that is in the midst of a hepatitis C epidemic that is directly related to increasing prescription opioid abuse and injection of heroin [48, 49]. Additionally, injection use appeared to be entirely self-reported in all studies, and there were variances in the length of time from last injection criterion between studies, with some studies enrolling individuals who reported ever injecting [40] to other studies only enrolling individuals who injected at least once in the previous month [43, 45].

### Variable interventions

Participation in NEP was self-reported in all studies. However, the frequency of attendance was highly variable between studies, ranging from questions querying whether or not a program had ever been used [40, 41] to NEP attendance at least once per week in the previous 6 months [43]. No pattern between study findings and reported frequency of attendance was observed.

### Variable outcomes

Although all studies included a serological measure of HCV, one study demonstrating a harmful effect used saliva measurements [44], which may vary in accuracy compared to serum tests. Additionally, the particular assay used in the serum measurements was variable with some studies using second-generation assays and others using third-generation assays.

### Variable study designs and analyses

The particular type of outcome assessed was directly related to variability in study design and the associated outcomes. In the current review, cohort studies that incorporated a time component into the analysis and reported hazard ratios, demonstrated a significant increase in the risk of HCV infection for NEP users; whereas, the lone case–control study [40] that reported an odds ratio demonstrated a highly significant preventative effect from NEP use. In contrast, Holtzman et al. [42] analyzed data from a combination of observational cohort studies and a subset of data from a randomized behavioral intervention, and reported an odds ratio that demonstrated no significant effect from NEP participation on the prevention of HCV. These differences in outcomes precluded the ability to combine all effect sizes into one model and instead resulted in two models with different interpretations. Although this plan differs from the combined model presented by Hagan et al. [12], we made this decision a priori based on the rationale that odds ratios and hazard ratios are two fundamentally different measures, given that the latter incorporates a time component (i.e., time-to-event data). Although not recommended, time-to-event data can sometimes be analyzed as dichotomous data that yield odds ratios [26]. However, such an analysis requires that the status (e.g., serostatus) of all patients be known at a fixed time point (i.e., 12 months) [26], which was not reported in all studies included in our hazard ratio model. Further complicating matters is that some studies made adjustments to the overall reported main outcome whereas other studies only reported unadjusted results. Among those studies that made adjustments, different potential confounders were controlled for likely leading to additional heterogeneity between studies.

In a recent review of reviews, MacArthur et al. [21] reported a similar pattern of variability in findings by study design. Of the 17 studies examining the impact of NEP on the prevention of HCV, nine studies (one case–control study, six cross-sectional studies, and two ecological studies) demonstrated a positive (or preventative) effect from NEP use. In contrast, two cohort studies demonstrated a negative (or harmful) effect. The remaining six studies demonstrated no association and were evenly split between three cohort studies and three cross-sectional studies.

While we made an a priori decision to exclude cross-sectional studies in our meta-analysis due to the fact that such designs can only assess associations between NEP participation and HCV infection, the current review identified five cross-sectional studies that met all other inclusion criteria [50–54]. Results from these studies were mixed with one study finding no effect from NEP participation (OR, 1.54, 95% CI, 0.73–3.24) [50], one study finding a preventative effect (OR, 0.59, 95% CI, 0.43–0.77) [53], and three studies demonstrating a harmful effect (OR, 2.17, 95% CI, 1.38–3.40 [51]; OR, 2.1, 95% CI, 1.54–2.89 [52]; OR, 2.54, 95% CI, 1.36–4.74 [54]). Similar to the findings from the current review, not all studies adjusted odds ratios for confounding, and the various adjustments made were not uniform between studies. Qualitative heterogeneity in the measurement of both the exposure (NEP use) and outcome (HCV infection) were also reported. Finally, Turner et al. [55] conducted a meta-analysis of cross-sectional and cohort studies conducted in the UK and observed no effect from NEP participation on HCV incidence (ES, 0.58, 95% CI, 0.30–1.15). Interestingly, and in contrast to all other studies, no inconsistency (*I* ^*2*^ = 0.0%) was reported in the model. The two cohort studies included in this model did not contain data that would allow calculation of the association between NEP use and HCV infection (inclusion criteria #6).

### HIV studies

The heterogeneity observed in this systematic review with meta-analysis is not limited to studies examining the effectiveness of NEP for the prevention of HCV. A recent systematic review with meta-analysis by Aspinall et al. [56] examining the influence of NEP on prevention of HIV observed large inconsistency (*I* ^*2*^ *= 7*5.7%) between the 12 included studies (10 cohort, 1 cross-sectional, and 1 case–control). Although a preventative effect from NEP exposure was suggested, the upper bound of the confidence interval slightly crossed 1 (pooled effect size, 0.66, 95% CI 0.42–1.01). When higher-quality studies, as graded by the Newcastle-Ottawa Scale, were combined, a significant preventative effect was observed (0.42, 95% CI, 0.22–0.81), although large inconsistency (*I* ^*2*^ = 80%) remained. Similar to the current review, variability in study populations, measurements of the intervention and exposure, and statistical analyses, likely contributed to significant heterogeneity in the models. In particular, the authors graded the overall quality of the evidence as “low” due to considerable limitations observed in the primary studies. Potential confounding of results from historical threats to internal validity, especially the introduction of antiretroviral medicines that minimize transmissible viral load and sexual health promotion programs, was a noted concern. Additionally, the statistical power to detect a significant result was low due to the fact that HIV seroconversions were a relatively rare event, which is also a problem in studies examining the role of NEP in preventing HCV seroconversion. Of note, many of the primary studies only examined HIV incidence as a secondary outcome.

### Implications for research and practice

The previously discussed qualitative between-study differences that may be contributing to the substantial statistical heterogeneity and large inconsistency raise several important implications for future research. More specifically, there is a need for well-designed cohort studies that follow seronegative individuals forward in time to track potential seroconversion. It is suggested that these studies seek standardization of interventions and outcomes in the following areas: (1) inclusion criteria, (2) injection use timeframe, (3) definition and measurement of NEP use, (4) outcome assessment, and (5) statistical analysis plan.

With regard to suggestion 3, more objective measures of program attendance are recommended, but may be difficult to implement in practice. In particular, some needle exchanges do not require identification to obtain needles [57], which precludes the ability to objectively track program attendance. Alternatively, a randomly assigned identification number could be implemented to track both program attendance and any potential seroconversion.

With regard to suggestion 5, given the fact that a recent meta-analysis found a 94% increased risk of HCV seroconversion among injection drug users who shared syringes (pooled risk ratio = 1.94, 95% confidence interval (CI) 1.53, 2.46) [58], the sharing of syringes is an important covariate that should be standardized, measured, and adjusted for in future studies. Although a recent report by the US Centers for Disease Control (CDC) suggested that NEPs can reduce the sharing of syringes, White PWIDs, who are largely driving the HCV epidemic in nonurban areas, had the highest rate of syringe sharing [59].

Importantly, merely providing clean needles to PWIDs may not be enough to prevent new cases of HCV. Crofts et al. [60] first documented new HCV infections in PWIDs who reported no needle sharing, which suggests that HCV infection could be spread in other ways, such as sharing of contaminated equipment (e.g., mixing spoons and filters). Furthermore, a recent study conducted with heroin injectors in Denver, Colorado, observed barriers to using a clean needle for every injection, such as being in withdraw and fear of arrest that may prevent the use of a clean needle for every injection [61]. However, research examining these barriers in rural settings is nonexistent and represents an area ripe for inquiry.

Given the mixed findings and substantial heterogeneity and inconsistency observed in both this review and previous reviews, there is insufficient empirical evidence to either recommend or discount NEP for the prevention of HCV. However, despite this mixed evidence, the US CDC recently recommended implementation of these programs in rural areas that have been disproportionately affected by the recent opioid and heroin epidemics [59]. Unfortunately, not much is currently known regarding the experience of opening these programs in rural areas. Therefore, research elucidating the unique context in which these programs are implemented in rural areas, as well as the challenges and barriers experienced, is needed. To the best of our knowledge, there are currently only nine programs listed in the North American Syringe Exchange Network that are located in Central Appalachia (two programs in Kentucky, one in Tennessee, none in Virginia, and six in West Virginia) [62]. However, this total represents eight additional programs that have opened since June, 2014, when only one program operated in Nashville Tennessee [63].

### Strengths

There are at least six potential strengths of the current meta-analysis. First, to the best of the author’s knowledge, this systematic review with meta-analysis represents the first work using meta-analytic methods to provide quantitative estimates of the impact of NEPs on the prevention of HCV in PWIDs since the work of Hagan et al. [12]. The recently published systematic review with meta-analysis by Sawangjit et al. [23] only focused on pharmacy-based NEP and HCV prevalence (as opposed to the prevention of incident cases). Secondly, included studies were limited to designs which promote drawing causal inferences (i.e., cohort and case–control). Third, studies were not limited to the injection of illegal drugs. Fourth, these mixed results are consistent with previous studies. Fifth, the use of an objective serological measure of the outcome minimized potential bias in the reporting of HCV status. Sixth, this review has led to specific recommendations for the design of studies to minimize between-study heterogeneity and inconsistency, which may be preventing definitive conclusions regarding the effect of NEPs on the prevention of HCV.

### Limitations

There are at least six potential limitations to be considered when reviewing the results of this meta-analysis. First, the current study excluded SIFs and pharmacies from the search due to our objective of examining the evidence related to programs that both collect and distribute (i.e., exchange) needles. In contrast, SIFs primarily provide clean needles for the injection of drugs on-site under medical monitoring. However, it has been noted that SIFs may have an important role in preventing HCV infection among PWIDs by serving as an additional mechanism for the provision of sterile needles in addition to NEPs [64]. Therefore, our results are limited to only one mechanism of sterile needle access. In addition to sterile needle provision, SIFs may greatly reduce risky injection practices (i.e., syringe sharing) that lead to HCV infection [64]. Unfortunately, SIFs are not yet widely available in the USA, in general, and in the rural areas of the USA that are in the midst of the HCV epidemic, in specific. The very first SIFs in the USA are preparing to open in 2017 in an urban location on the West Coast of the USA [65]. Current evidence regarding their impact on HCV seroconversion is lacking. Hagan et al. [12], upon which we based our search strategy, failed to find any articles describing the impact of SIFs or pharmacy sales on HCV seroconversion that met their inclusion criteria for their systematic review with meta-analysis. There were 15 studies among the 555 included in this systematic review that described SIFs. Only two of these studies obtained an objective measure (i.e., serum or saliva) of HCV infection. However, both studies were cross-sectional and did not provide data that would allow calculation of the association between SIF use and HCV infection. Similarly, and as discussed above, there is very limited evidence (*N* = 2 studies) that currently exists on the impact of pharmacy-based NEP, an area ripe for further inquiry. Secondly, the current review was unable to formally assess differential study effects stemming from different designs using moderator analysis due to the small sample size. Third, the weaknesses and potential biases inherent in individual studies are included in a meta-analysis, which may have negatively affected this study’s ability to detect significant results. Such biases include information bias that could have been present from self-reports of injection status and NEP attendance, as well as volunteer bias [66, 67], which represents the phenomenon of NEP attendance by PWIDs that may be at higher risk for infectious disease. Fourth, it is possible that studies were missed during the systematic review and not included in the meta-analysis. In addition to not searching for unpublished sources, the fact that over 100 full text articles had to be reviewed to assess inclusion and exclusion criteria may indicate that studies that address this topic are not well described in either the title or abstract. Fifth, a small number of included studies precluded a complete assessment of the possibility of small study effects, including publication bias. Finally, because the aggregate data approach for this meta-analysis was used, these results are subject to ecological fallacy [68].

## Conclusions

The impact of NEP on the prevention of HCV in PWIDs remains unclear. Such lack of clarity is likely due to substantial between-study heterogeneity in study design, inclusion criteria, intervention definition, outcome assessment, and statistical analyses that yield different pooled results depending on whether or not a time component (hazard ratio) is included in the analysis. Studies examining the operation of NEPs in rural areas are particularly needed, along with research examining the unique barriers to using clean needles experienced by PWIDs, to clarify the overall contribution of the presence of clean needles in the environment obtained from NEPs to the successful prevention of new cases of HCV. Future studies should also examine the impact of other sources of clean needles available for injection, such as SIFs and pharmacies, on the prevention of HCV infection in PWIDs. Given the potential benefits of NEP for reducing infectious disease in a population, future studies incorporating standardized populations, interventions, comparisons, outcomes, and analyses are critically needed to inform public health practice and policy.

## Additional files

Additional file 1: Search strategy examples. This document contains the queries entered into each database. (PDF 236 kb)

Additional file 2: Screening list and codebook. This spreadsheet contains the reasons for inclusion and exclusion for all screened abstracts and articles, and the codebook for included articles. (XLSX 147 kb)

## Abbreviations

HCV: Hepatitis C virus
HIV: Human immunodeficiency virus
HR: Hazard ratio
NEP: Needle exchange program
NNR: Number needed to read
NOS: Newcastle-Ottawa Quality Assessment Scale
OR: Odds ratio
PRISMA: Preferred Reporting Items for Systematic Reviews and Meta-analyses
PWID: People who inject drugs
SIF: Supervised injection facility
