# Supplementary material for: Needle exchange programs for the prevention of hepatitis C virus infection in people who inject drugs: a systematic review with meta-analysis
Source: Harm Reduct J. 2017 May 17;14:25. doi: 10.1186/s12954-017-0156-z (PMC5436422; doi:10.1186/s12954-017-0156-z)
Supplement: Supplementary file 1 — Search strategy examples. This document contains the queries entered into each database. (PDF 236 kb) [file 12954_2017_156_MOESM1_ESM.pdf]

Search Details

PubMed Search 07/18/2016
**Result:**
314
**Stopword(s) Ignored:**
use
**Translations:**

hepatitis C	"hepatitis c"[MeSH Terms] OR "hepatitis c"[All Fields] OR "hepacivirus"[MeSH Terms] OR "hepacivirus"[All Fields]
intravenous drug abuse	"substance abuse, intravenous"[MeSH Terms] OR ("substance"[All Fields] AND "abuse"[All Fields] AND "intravenous"[All Fields]) OR "intravenous substance abuse"[All Fields] OR ("intravenous"[All Fields] AND "drug"[All Fields] AND "abuse"[All Fields]) OR "intravenous drug abuse"[All Fields]
drug misuse	"drug users"[MeSH Terms] OR ("drug"[All Fields] AND "users"[All Fields]) OR "drug users"[All Fields] OR

	("drug"[All Fields] AND "misuse"[All Fields]) OR "drug misuse"[All Fields]
drug addict	"drug users"[MeSH Terms] OR ("drug"[All Fields] AND "users"[All Fields]) OR "drug users"[All Fields] OR ("drug"[All Fields] AND "addict"[All Fields]) OR "drug addict"[All Fields]
drug abuse	"substance-related disorders"[MeSH Terms] OR ("substance-related"[All Fields] AND "disorders"[All Fields]) OR "substance-related disorders"[All Fields] OR ("drug"[All Fields] AND "abuse"[All Fields]) OR "drug abuse"[All Fields]
drugs	"pharmaceutical preparations"[MeSH Terms] OR ("pharmaceutical"[All Fields] AND "preparations"[All Fields]) OR "pharmaceutical preparations"[All Fields] OR "drugs"[All Fields]
people	"persons"[MeSH Terms] OR "persons"[All Fields] OR "people"[All Fields]
prevention	"prevention and control"[Subheading] OR ("prevention"[All Fields] AND "control"[All Fields]) OR "prevention and control"[All Fields] OR "prevention"[All Fields]
risk factor	"risk factors"[MeSH Terms] OR ("risk"[All Fields] AND "factors"[All Fields]) OR "risk factors"[All Fields] OR ("risk"[All Fields] AND "factor"[All Fields]) OR "risk factor"[All Fields]

epidemiology	"epidemiology"[Subheading] OR "epidemiology"[All Fields] OR "epidemiology"[MeSH Terms]
prevalence	"epidemiology"[Subheading] OR "epidemiology"[All Fields] OR "prevalence"[All Fields] OR "prevalence"[MeSH Terms]
incidence	"epidemiology"[Subheading] OR "epidemiology"[All Fields] OR "incidence"[All Fields] OR "incidence"[MeSH Terms]
seroprevalence	"seroepidemiologic studies"[MeSH Terms] OR ("seroepidemiologic"[All Fields] AND "studies"[All Fields]) OR "seroepidemiologic studies"[All Fields] OR "seroprevalence"[All Fields]
seroconversion	"seroconversion"[MeSH Terms] OR "seroconversion"[All Fields]
genotype	"genotype"[MeSH Terms] OR "genotype"[All Fields]
exchange	"Sex Health Exch"[Journal] OR "exchange"[All Fields]
needle	"needles"[MeSH Terms] OR "needles"[All Fields] OR "needle"[All Fields]
needle exchange program	"needle-exchange programs"[MeSH Terms] OR ("needle-exchange"[All Fields] AND "programs"[All Fields]) OR "needle-exchange programs"[All Fields] OR ("needle"[All

	Fields] AND "exchange"[All Fields] AND "program"[All Fields]) OR "needle exchange program"[All Fields]
syringe exchange program	"needle-exchange programs"[MeSH Terms] OR ("needle-exchange"[All Fields] AND "programs"[All Fields]) OR "needle-exchange programs"[All Fields] OR ("syringe"[All Fields] AND "exchange"[All Fields] AND "program"[All Fields]) OR "syringe exchange program"[All Fields]
syringe	"syringes"[MeSH Terms] OR "syringes"[All Fields] OR "syringe"[All Fields]

Database:

PubMed

User query:

(hepatitis C OR HCV) AND (intravenous drug abuse OR intravenous drug use OR drug misuse OR drug addict OR injecting drug use OR drug abuse OR people who inject drugs OR IDU OR PWID) AND (prevention OR risk factor OR epidemiology OR prevalence OR incidence OR seroprevalence OR seroincidence OR seroconversion OR genotype OR coinfect*) AND (needle exchange OR needle exchange program OR syringe exchange program OR syringe access program) AND ("1989/01/01"[Date - Publication] : "3000"[Date - Publication])

Scopus Query Details 7/18/2016

Scopus refine results values

Your query : ((TITLE-ABS-KEY("hepatitis C") OR TITLE-ABS-KEY("HCV") AND TITLE-ABS-KEY("intravenous drug abuse") OR TITLE-ABS-KEY("intravenous drug use") OR TITLE-ABS-KEY("drug misuse") OR TITLE-ABS-KEY("drug addict") OR TITLE-ABS-KEY("injecting drug use") OR TITLE-ABS-KEY("drug abuse") OR TITLE-ABS-KEY("people who inject drugs") OR TITLE-ABS-KEY("IDU") OR TITLE-ABS-KEY("PWID") AND TITLE-ABS-KEY("prevention") OR TITLE-ABS-KEY("risk factor") OR TITLE-ABS-KEY("epidemiology") OR TITLE-ABS-KEY("prevalence") OR TITLE-ABS-KEY("incidence") OR TITLE-ABS-KEY("seroprevalence") OR TITLE-ABS-KEY("seroincidence") OR TITLE-ABS-KEY("seroconversion") OR TITLE-ABS-KEY("genotype") OR TITLE-ABS-KEY("coinfect*") AND TITLE-ABS-KEY("needle exchange") OR TITLE-ABS-KEY("needle exchange program") OR TITLE-ABS-KEY("syringe exchange program") OR TITLE-ABS-KEY("syringe access program")) AND PUBYEAR > 1988)

Number of results : 226

Searched 8-18-16

WEB OF SCIENCE™

THOMSON REUTERS™

- Search
- My Tools
- Search History

Search History: All Databases

Set	Results	Save History / Open Saved History	Combine Sets (AND / OR) Combine	Delete Sets (Select All / Delete)
# 1	511	**TOPIC:** ((((hepatitis C OR HCV) AND (intravenous drug abuse OR intravenous drug use OR drug misuse OR drug addict OR injecting drug use OR drug abuse OR people who inject drugs OR IDU OR PWID) AND (prevention OR risk factor OR epidemiology OR prevalence OR incidence OR seroprevalence OR seroincidence OR seroconversion OR genotype OR coinfect*) AND (needle exchange OR needle exchange program OR syringe exchange program OR syringe access program))))<br>*Timespan=1989-2016*<br>*Search language=Auto*	Select to combine sets.	Select to delete this set.
			AND OR Combine	Select All Delete

© 2016 THOMSON REUTERS

- TERMS OF USE
- PRIVACY POLICY
- FEEDBACK

CINHAL Search 7/18/16

S1	(hepatitis C OR HCV) AND (intravenous drug abuse OR intravenous drug use OR drug misuse OR drug addict OR injecting drug use OR drug abuse OR people who inject drugs OR IDU OR PWID) AND (prevention OR risk factor OR epidemiology OR prevalence OR incidence OR seroprevalence OR seroincidence OR seroconversion OR genotype OR coinfect*) AND (needle exchange OR needle exchange program OR syringe exchange program OR syringe access program)	**Search modes** - Find all my search terms

Number of Results: 102
